# 

**Published:** 1890-05-10

**Authors:** 


Registered at the General Post Office as a Newspaper for transmission in the United Kingdom and Abroad.

Per Annum, 6s. 6d.; post free.
Monthly Parts, 6d.; post free 8d.
Ditto per Annum, 8s., post free.

### THE HOSPITAL

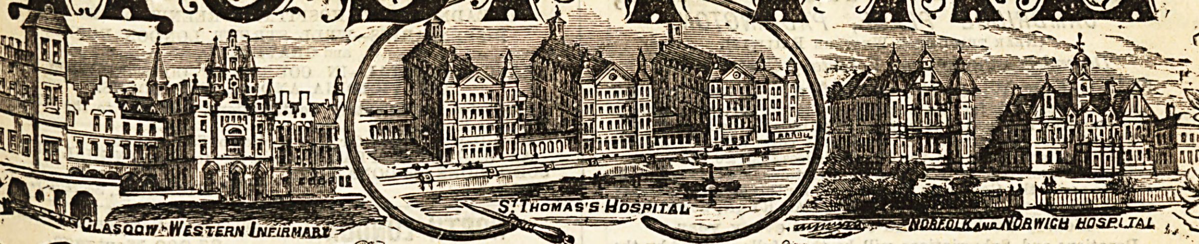

A WEEKLY JOURNAL OF

### Science, Medicine, Nursing and Philanthropy.

SATURDAY, MAY 10th, 1890.

